# Genome-wide characterization of the *NLR* gene family in tomato (*Solanum lycopersicum*) and their relatedness to disease resistance

**DOI:** 10.3389/fgene.2022.931580

**Published:** 2022-12-05

**Authors:** Sehrish Bashir, Nazia Rehman, Fabia Fakhar Zaman, Muhammad Kashif Naeem, Atif Jamal, Aurélien Tellier, Muhammad Ilyas, Gustavo Adolfo Silva Arias, Muhammad Ramzan Khan

**Affiliations:** ^1^ National Institute for Genomics and Advanced Biotechnology, National Agricultural Research Centre, Islamabad, Pakistan; ^2^ PARC Institute for Advanced Studies in Agriculture, NARC, Islamabad, Pakistan; ^3^ Crop Disease Research Institute, National Agricultural Research Center, Islamabad, Pakistan; ^4^ Population Genetics, Department of Life Science Systems, School of Life Sciences, Technical University of Munich, Freising, Germany

**Keywords:** *NLR* genes, phylogenetic relationship, synteny, *Alternaria solani*, *Phytophthora infestans*, tomato

## Abstract

Nucleotide-binding leucine-rich-repeat receptors (NLR), the largest group of genes associated with plant disease resistance (R), have attracted attention due to their crucial role in protecting plants from pathogens. Genome-wide studies of NLRs have revealed conserved domains in the annotated tomato genome. The 321 *NLR* genes identified in the tomato genome have been randomly mapped to 12 chromosomes. Phylogenetic analysis and classification of NLRs have revealed that 211 genes share full-length domains categorized into three major clades (CNL, TNL, and RNL); the remaining 110 NLRs share partial domains and are classified in CN, TN, and N according to their motifs and gene structures. The cis-regulatory elements of NLRs exhibit the maximum number of these elements and are involved in response to biotic and abiotic stresses, pathogen recognition, and resistance. Analysis of the phylogenetic relationship between tomato NLRs and orthologs in other species has shown conservation among Solanaceae members and variation with *A. thaliana*. Synteny and Ka/Ks analyses of *Solanum lycopersicum* and *Solanum tuberosum* orthologs have underscored the importance of *NLR* conservation and diversification from ancestral species millions of years ago. RNA-seq data and qPCR analysis of early and late blight diseases in tomatoes revealed consistent *NLR* expression patterns, including upregulation in infected compared to control plants (with some exceptions), suggesting the role of NLRs as key regulators in early blight resistance. Moreover, the expression levels of NLRs associated with late blight resistance (*Solyc04g007060 [NRC4]* and *Solyc10g008240 [RIB12]*) suggested that they regulate *S. lycopersicum* resistance to *P. infestans*. These findings provide important fundamental knowledge for understanding *NLR* evolution and diversity and will empower the broader characterization of disease resistance genes for pyramiding through speed cloning to develop disease-tolerant varieties.

## Introduction

Solanaceae is a diverse plant family that contains economically important crops including potato (*Solanum tuberosum*), tomato (*Solanum lycopersicum*), tobacco (*Nicotiana attenuata*), and pepper (*Capsicum annum*) (Särkinen et al., 2013). Tomato is the most valuable cash crop after potato. Its growth and yield production are hindered by various biotic and abiotic factors. Among biotic factors, multiple pathogen invasions, including fungi, oomycetes, bacteria, viruses, and nematodes, cause a 15% harvest reduction, ultimately affecting national economies (Narusaka et al., 2018). However, tomato plants are susceptible to >200 diseases that can affect the plants at any growth stage. Tomatoes are mainly affected by fungal pathogens including *Phytophthora infestans* and *Alternaria solani*, which cause late and early blight disease, respectively (Qian et al., 2017). These fungal pathogens collectively accounted for 49–91% of tomato yield loss in Pakistan (Vleeshouwers et al., 2011).

Plants have evolved elaborate methods for immunity against pathogen invasion while initiating signaling pathways through the defense layer mechanism (Seo et al., 2016). This mechanism is regulated by cell surface barrier and pattern recognition receptors, which provide conserved pathogen-associated molecular patterns (PAMPs) for broad-spectrum resistance. Moreover, a second defensive layer is driven by intracellular immune receptors that induce effector-triggered immunity (ETI). Most intracellular immune receptors in plants belong to the resistance gene (R-gene) family, which plays a key role in shielding against pathogens and programmed cell death (Qian et al., 2017). The R-gene family includes transmembrane proteins such as receptor-like kinases (RLKs) and receptor-like proteins (RLPs) and kinase-like proteins; i.e., the Pto-gene family and nucleotide-binding-leucine-rich repeat receptor (NLR)-encoding gene family (Andolfo et al., 2013).

A large NLR gene family in the tomato genome has been identified and re-annotated using advanced techniques (Andolfo et al., 2014). These NLRs act as signaling networks by inducing pathogenesis-related proteins, producing an apoptotic hypersensitive response and conferring resistance by recognizing pathogen-effector proteins (Seong et al., 2020). The conserved component of the NLRs is NB-ARC, which comprises a nucleotide-binding site (NB), Apaf-1 (apoptotic protease activating factor 1), resistance proteins (R), and CED4 (cell death protein-4) (Meyers et al., 2003). NLRs are also classified based on multi-domain characterization and further diversified by their N- and C-terminal regions. The N-terminus of the Toll-like/interleukin 1 domain is a TIR-type NLR that initiates downstream signaling through enhanced disease susceptibility 1 (ESD1). Additionally, the coiled-coil domain at the N-terminus causes non-race-specific disease resistance 1 (NDR1) (McHale et al., 2006). A small proportion of *NLR* proteins show N-terminus resistance to powdery mildew 8 (RPW8) domains (RNL) (Shao et al., 2014). Another variable region, the LRR domain at the C-terminus, makes the full-length gene sequences TNL, CNL, and RNL, while some genes lack and encode the partial domain sub-families CN, TN, and N. Both variable *NLR* regions exhibit specificity toward pathogen recognition by interacting with specific ligands to introduce resistance and are implicated in the activation of corresponding pathways (Shi et al., 2021). Moreover, *NLR* required for cell death (NRC) works as a helper to NLRs, which evolve *via* duplication and establish specificities to diverse pathogens together with sensor NLRs (Wu et al., 2017).

To date, more than 300 R-genes have been cloned from a wide range of plant species for resistance. Among these, 80% of genes encode *NLR* proteins (Kourelis and Van Der Hoorn, 2018). Twenty NLRs against various pathogens have been identified and cloned within the Solanaceae family. Previous genome-wide studies identified NLRs in tomatoes through a conserved domain framework, providing insight into their evolution and diversification in different sub-families (Vossen et al., 2016). Recent improvements in the tomato genome annotation and the use of the RenSeq technique have provided more information to annotate and characterize many genes and also identify novel genes. Therefore, the present study applied a domain-based framework with genome-wide analyses to identify the new NLRs and understand their genetic role in disease resistance. The findings of this study may enhance the understanding of the dynamic role of *NLR* resistance against invading pathogens based on their evolutionary relationships and analyses of their promoters. Furthermore, we validated the *NLR* genes by qPCR and MQTLs to inform the development of new varieties through gene expression regulation and speed cloning based on the pyramiding of resistance genes to achieve durable tolerance.

## Materials and methods

### Identification of NLRs orthologs, motifs, and gene structures and phylogenetic analyses

BlastP was performed using the protein sequences of previously reported domains of the *NLR* gene family as the query sequences (van der Biezen and Jones, 1998; Meyers et al., 1999; Xiao et al., 2001). To identify the *NLR* orthologs in the tomato genome, Ensembl Plants (https://plants.ensembl.org/index.html) (Bolser et al., 2016) and Sol Genomics Network (SGN) (https://solgenomics.net/) were used. The sequences were retrieved and confirmed in the ANNA database (https://biobigdata.nju.edu.cn/ANNA/) (Liu et al., 2021). After removing duplicate genes, the tomato *NLR* genes were evaluated for sequence-specific domains using the SMART tool (http://smart.embl-heidelberg.de/) (Schultz et al., 1998), conserved domain database (https://www.ncbi.nlm.nih.gov/Structure/cdd/wrpsb.cgi) (Marchler-Bauer et al., 2002), and Pfam domain (https://pfam.xfam.org/) (Finn et al., 2003). After filtering the sequences based on conserved domains, 321 *NLR* genes were identified using a different pipeline from earlier data (Andolfo et al., 2014; Seo et al., 2016; Seong et al., 2020). MEME v.5.4.1 was used to predict the specific conserved motifs in 321 tomato NLRs (Bailey et al., 2009). Gene structure display server (GSDS2.0) (http://gsds.gao-lab.org/) was used to draw the gene structures (Hu et al., 2015). To understand the diversity and relationship among NLRs within the tomato genome, the protein sequences were subjected to multiple sequence alignment using CLUSTALW (https://www.genome.jp/tools-bin/clustalw) and a maximum likelihood (ML) tree with 1000 bootstrap replications for intact and partial domain protein sequences in MEGAX (https://www.megasoftware.net/). The comprehensive demonstration of the motifs and gene structure diversity of the sequences present in the same clades of the phylogenetic tree were visualized using TBtools (Chen et al., 2018). The molecular and structural features of tomato NLRs were calculated using EXPASY ProtParam (https://web.expasy.org/protparam/) (Gasteiger et al., 2005). Sub-cellular localization of the identified NLRs was determined using CELLO v.2.5 (http://cello.life.nctu.edu.tw/) (Yu et al., 2006).

### Evolutionary relationships between tomato *NLR* genes and other species

BlastP was performed using tomato *NLR* protein sequences on the Ensembl Plants database against *Solanum tuberosum, Nicotiana attenuata, Capsicum annum, Arabidopsis thaliana*, and the outlier *Saccharomyces cerevisiae* to identify their orthologs. A threshold of identity >70% and E-value <1e^-5^ identified 16 orthologs from *A. thaliana*, 34 from *S. tuberosum*, 27 from *C. annum*, 14 from *N. attenuata*, and two from *S. cerevisiae*. A phylogenetic tree was constructed using MEGAX (https://www.megasoftware.net/) through the ML method with 1000 bootstrap replications (Kumar et al., 2018). The final tree annotation was produced using the online iTOL (Interactive Tree of Life) (https://itol.embl.de/) (Letunic and Bork, 2016).

### Promoter analysis

Cis-regulatory elements identified in the promoter region of *NLR* genes belonged to nine different sub-classes. Regions 1500 bps upstream of the selected tomato gene IDs were downloaded from the Ensembl Plants database and submitted to the PlantCare online tool (http://bioinformatics.psb.ugent.be/webtools/plantcare/html/) (Lescot et al., 2002). The resultant values for cis-elements were visualized as a heatmap in TBtools.

### Synteny and Ka/Ks analysis

Protein and GFF3 files of tomato were retrieved from the Ensembl Plants database, while potato proteome and gene GFF3 files were obtained from the Sol Genomics Network (SGN) (https://solgenomics.net/). BlastP was performed for collinearity prediction. Syntenic gene pairs were identified and visualized as an advanced circos plot in TBtools. The protein and coding sequences of syntenic gene pairs between tomato and potato were acquired in a file for non-synonymous per synonymous (Ka/Ks) substitution rate of genes under selective pressure. The divergence time was calculated for evolutionary origin as T = Ks/2r, where x = 1.5 × 10^–8^ (Wai et al., 2021).

### RNA-seq data analysis

Transcriptomic RNA-seq data of early blight resistance in tomatoes were retrieved from the GEO database under accession number GSE75923 (Sarkar et al., 2017). Raw RNA-seq data on late blight were downloaded from GenBank Sequence Read Archive (SRA, SRP041501) (Zuluaga et al., 2016) to estimate the expression levels. The raw reads were mapped to the reference genome using HISAT2 version 2.1.0. StringTie version 2.1.5 was used to quantify the BAM and GTF files. The Ballgown package in R was used to generate an expression profile in FPKM (fragment per kilobase per million fragments mapped reads). Finally, a heatmap was produced using the FPKM values of *NLR* differentially expressed genes (DEGs) in TBtools.

### Plant materials and fungal disease inoculation

Four tomato (*S. lycopersicum*) genotypes (38037, 38046, 19890, and ROMA) and one wild *S. chilense* genotype (19906) were collected from the BCI (BioResources Conservation Institute), NARC (National Agriculture Research Center), Islamabad. Seeds were first sowed in trays under controlled conditions (glasshouse) at 22–25°C and 90% humidity. After 15 days, the seedlings were transferred to pots. The plants were inoculated with 30 ul of prepared fungal culture suspensions of *Alternaria solani* (1×10^3^ per ml) and conidial suspensions of *Phytophthora infestans* (5×10^4^ sporangia per ml). The inoculated plants were incubated in a moist growth chamber for 5–7 days at 22–28°C for *A. solani* and 18°C for *P. infestans* with 88–90% relative humidity (Arafa et al., 2017; Akhtar et al., 2019). The disease effects on the infected leaves were estimated as the percentage of affected area (Moghaddam et al., 2019).

### RNA extraction, cDNA synthesis, and quantitative real-time PCR

Leaf samples of control and disease-infected tomato plants were collected 7 days after infection. Total RNAs were extracted using an Invitrogen Pure Link™ RNA Mini Kit according to the manufacturer’s instructions. First-strand cDNA libraries were constructed from 0.5 µg of total RNA by using a Thermo Scientific Revert Aid Reverse Transcriptase kit (Scientific, 2000). For expression analysis, primers for selected upregulated and downregulated *NLR* genes (RPP13, RPM1, R1B12, R1B16, NRC4, and Rpi-gene) and an internal reference gene elongation factor (EF) were used (Supplementary Table S1). The 10-ul real-time PCR reactions (Applied Biosystems StepOnePlus™) included Maxima SYBR Green qPCR master mix, primers, and cDNA (control and infected samples) as a template. The assay was carried out in triplicate and data were analyzed.

### Chromosomal localization and meta-QTL analysis

The co-localization of NLRs with previously reported QTL regions linked to early and late blight resistance was observed using the meta-QTL approach and related QTLs in tomato retrieved from previous studies (Foolad et al., 2002; Zhang et al., 2003; Chaerani et al., 2007; Chen et al., 2014; Haggard et al., 2015; Mazumdar et al., 2021). NLRs and QTL distributions on the tomato chromosomes were visualized using MapChart (Voorrips, 2002). Early- and late blight-resistant QTLs linked to different NLRs were represented in different colors.

## Results

### Identification and evaluation of the *NLR* gene family in tomato

A sequence similarity search was performed in BlastP using *NLR* protein domain sequences (CNL, TNL, and RNL) as the query. Approximately 455 *NLR* homologs in tomato were identified according to the threshold (>50% identity and E-value <1e^-5^). The retrieved sequences were subjected to duplicate removal. After filtration, the sequences were submitted to the SMART, Pfam, and CDD databases to confirm the presence of *NLR* domains. A total of 321 *NLR* genes were screened for conserved domains reported in the Pfam database (NB-ARC: PF00931 and LRR: PF00560). This result was consistent with that of previous studies (Andolfo et al., 2014; Andolfo et al., 2021) based on RenSeq. Therefore, the improvement in the present protein domain-based BlastP pipeline is the description of the number of *NLR* genes and their classification according to the variable parts of the domain. Finally, the 321 *NLR* genes were divided into three major subfamilies within the *S. lycopersicum* genome and further categorized according to the domain as intact and partial domain-comprising genes (Supplementary Figure S1). These sequences included 256 CNLs (123 CNL, 35 genes are CNL-type CC-NB, 25 NLcc genes, 57 genes sharing the NB-ARC domain and 16 LRR only), 62 TNLs (21 TNL, 18 genes in TNL-type TIR-NB, and 23 NLtir) and three genes (*Solyc02g090380 (NRG1), Solyc04g079420 (ADR1)*, and *Solyc02g077060*) in the RNL sub-family (Supplementary Table S2). The 321 identified *NLR* genes in tomato with conserved domains were encoding proteins ranging from 64 to 2871 amino acid residues, gene sizes ranging from 194 to 16548 bps, molecular weights ranging from 3330 to 28617 kDa, isoelectric points ranging from 4.5 to 10.27, instability index varying from 10 to 70, and aliphatic indexes varying from 64 to 139 (Supplementary Table S3). Cellular localization determined that the genes were randomly distributed inside the cell independent of chromosome number. For instance, genes present on chromosome 1 were observed in the cytoplasm, plasma membrane, extracellular domain, and nucleus.

### Phylogenetic tree, motif, and gene structure analyses of NB domain-containing NLRs in tomato

**FIGURE 1:**
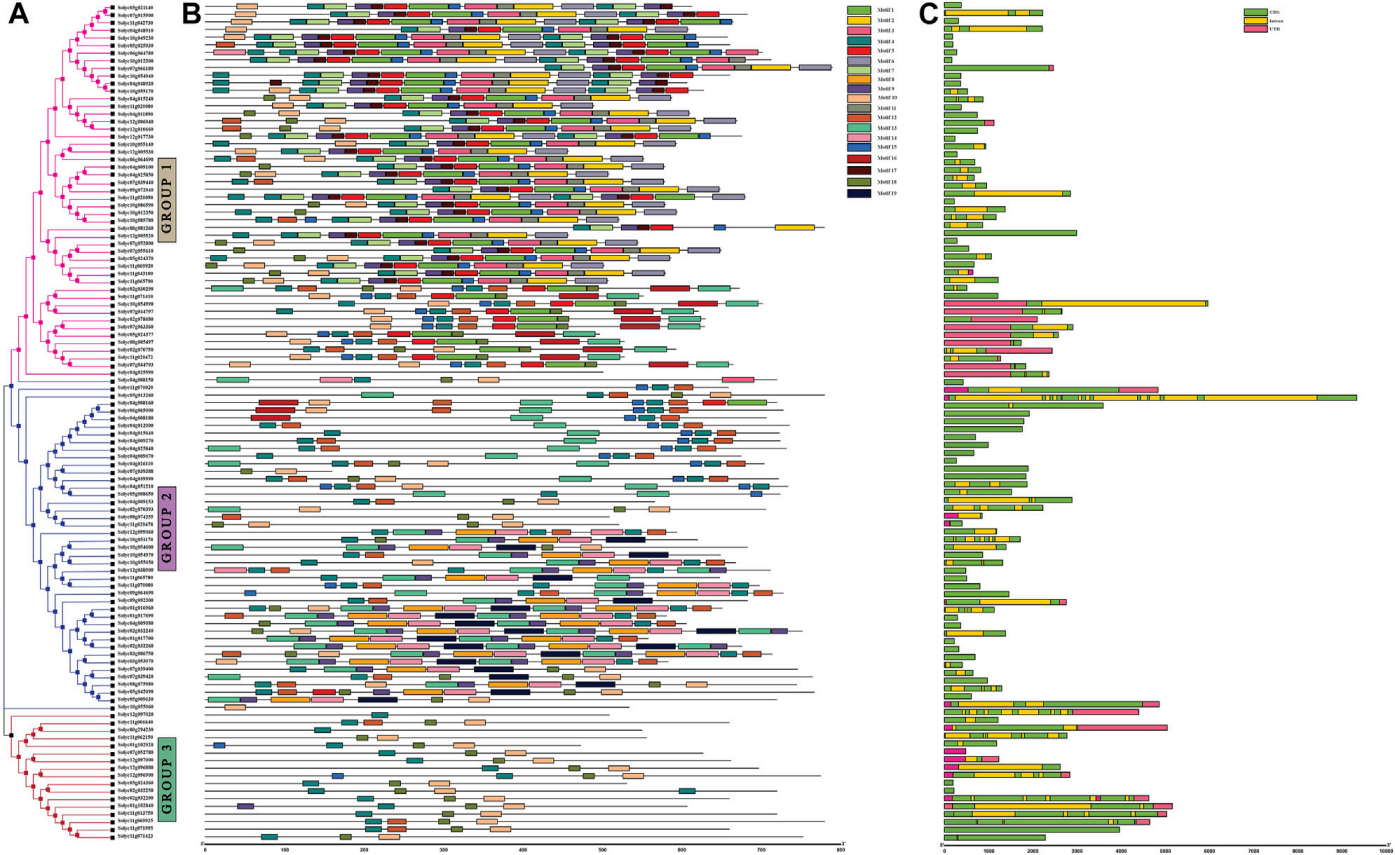
Rectangular tree image with motifs and gene structure analyses from TBtools. **(A)** Phylogenetic tree of *NLR* genes sharing partial domain (NB-coding genes) constructed in MEGAX using the maximum likelihood (ML) method with 1000 bootstrap replications. **(B)** Conserved motif composition/patterns represented in different colors generated using the MEME tool. **(C)** Gene structure analysis by GSDS.2.0. Green: CDS, pink: UTRs, yellow: introns.

A phylogenetic tree of the 110 identified partial-domain NB-coding protein sequences was constructed using MEGAX to determine the phylogenetic relationship of NB domain-containing genes in tomato. The genes were clustered into three groups. The first group represented NB-ARC domain-containing sequences, the second group included CC-NB-containing protein sequences, and the third group showed TIR-NB domain sequences (Figure 1A). The 110 NB-coding protein sequences were then subjected to MEME for analysis of 19 conserved motif patterns (Figure 1B). Major (P-loop, GLPL, and Kinase2) and minor (RNBS-B and RNBS-D) motifs were evaluated in the conserved part of the NB-ARC domain (Supplementary Figure S2). Overall, motifs 13 and 15 were conserved in most of the coding sequences, shown as part of their domains. We observed diversity in the types and numbers of motifs in the genes belonging to different sub-families. The same motif composition was observed in sequences in the same group; for instance, genes with the CC-NB domain showed the same motifs according to their domain profiles. TIR-NB- and NB-domain-containing sequences also represented the conserved motif composition.

The gene structures of the NB-coding sequences were analyzed using GSDS, which determined the number of introns, CDS, and UTRs (Figure 1C). Analysis of gene length variation and structures showed that *Solyc05g013260* contained the maximum of ten introns, while seven introns were present in *Solyc12g097020* in the TN sub-class. Five introns were observed in *Solyc10g054600* and *Solyc12g096900* from the NB and TN subclasses, respectively. Two genes from CN, seven genes from N, and two genes from TN showed at least one intron. Forty-four NB-coding genes had no intron and contained only CDS. The remaining sequences showed one or two UTR regions with one CDS and intron.

### Phylogenetic relationship, motif, and gene structure analyses of *NLR* domain-containing genes in tomato

**FIGURE 2:**
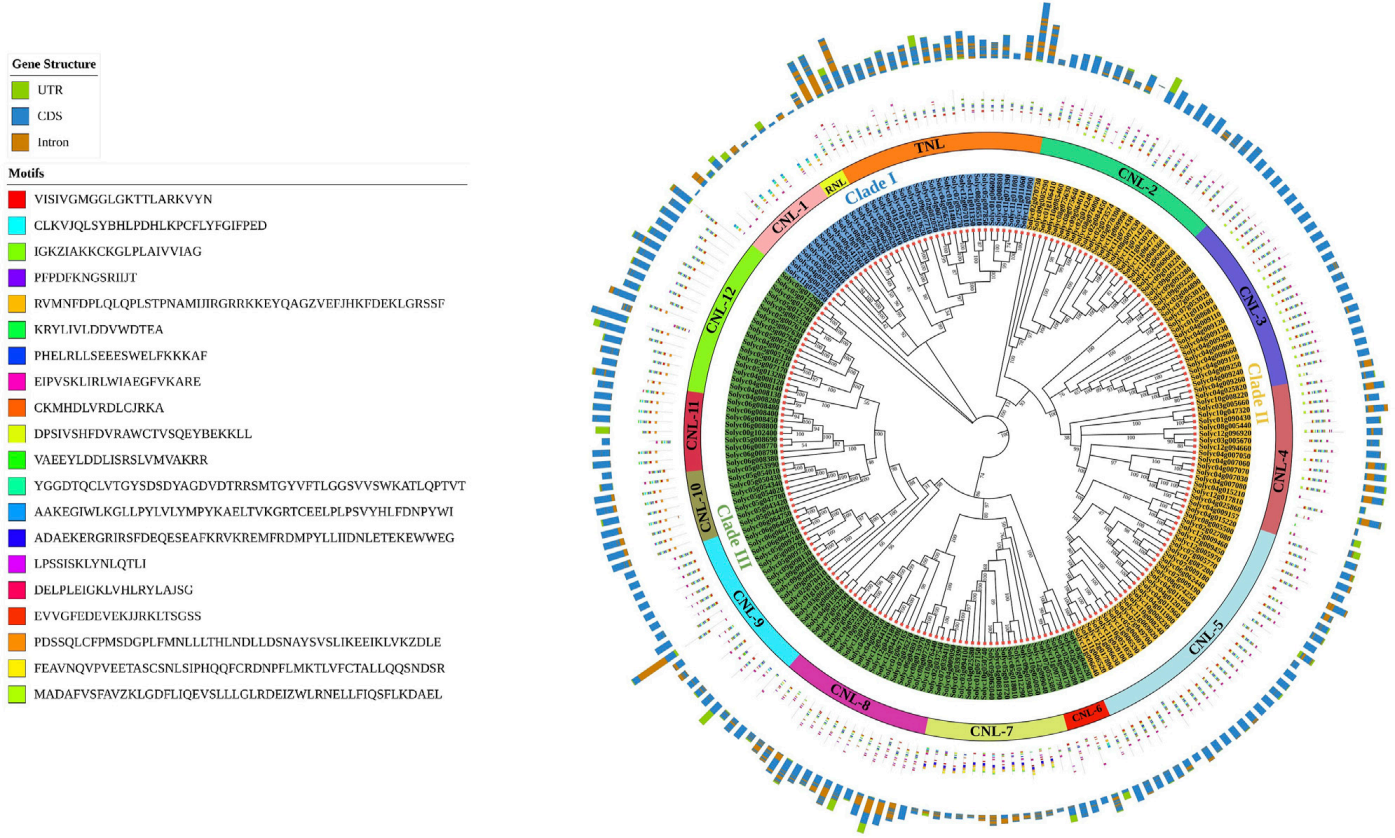
Phylogenetic tree within the tomato genome constructed using MEGAX and the maximum likelihood (ML) method with bootstrap replications after MSA in CLUSTALW. The evolutionary relationships among *NLR* genes containing full-length domains (three-sub families; CNL, TNL, and RNL) are shown. Brown: introns; blue: CDS; parrot: UTRs. Motif analysis was performed for 20 conserved motifs shown in different colors according to the legend. The CNL subfamily out-grouping into twelve subclusters (CNL G1–12) based on physical clustering is represented by different colors.

A total of 211 genes containing full-length CC-NB-LRR, TIR-NB-LRR, and RPW8-NB-LRR domains were subjected to phylogenetic analysis after multiple sequence alignment through CLUSTALW. The results showed that these genes were classified into three major clades and further subdivided into fourteen groups based on sequence similarities with the conserved domain. Clade I contained genes sharing the CNL-1, RNL, and TNL domains. Clade II shared four CNL gene distribution subgroups (CNL-2 to CNL-5). Clade III included seven CNL subgroups (CNL-6 to CNL-12). The CNL sub-class (CNL1-12) out-grouped the numbers of genes present in the other two groups as most of the genes fell into the CNL subfamily and made separate subclasses (Figure 2). Furthermore, the motif patterns were the same for the genes present in each subclade, except for *Solyc11g071390* and *Solyc11g042750*, which had no conserved motifs. Gene structure analysis of the 211 full-length domain sequences showed that 43 tomato genes had only exons and no introns, whereas *Solyc01g102880* contained the maximum number of introns (17).

### Analysis of the cis-regulatory elements in the promoter regions of tomato *NLR* genes

**FIGURE 3:**
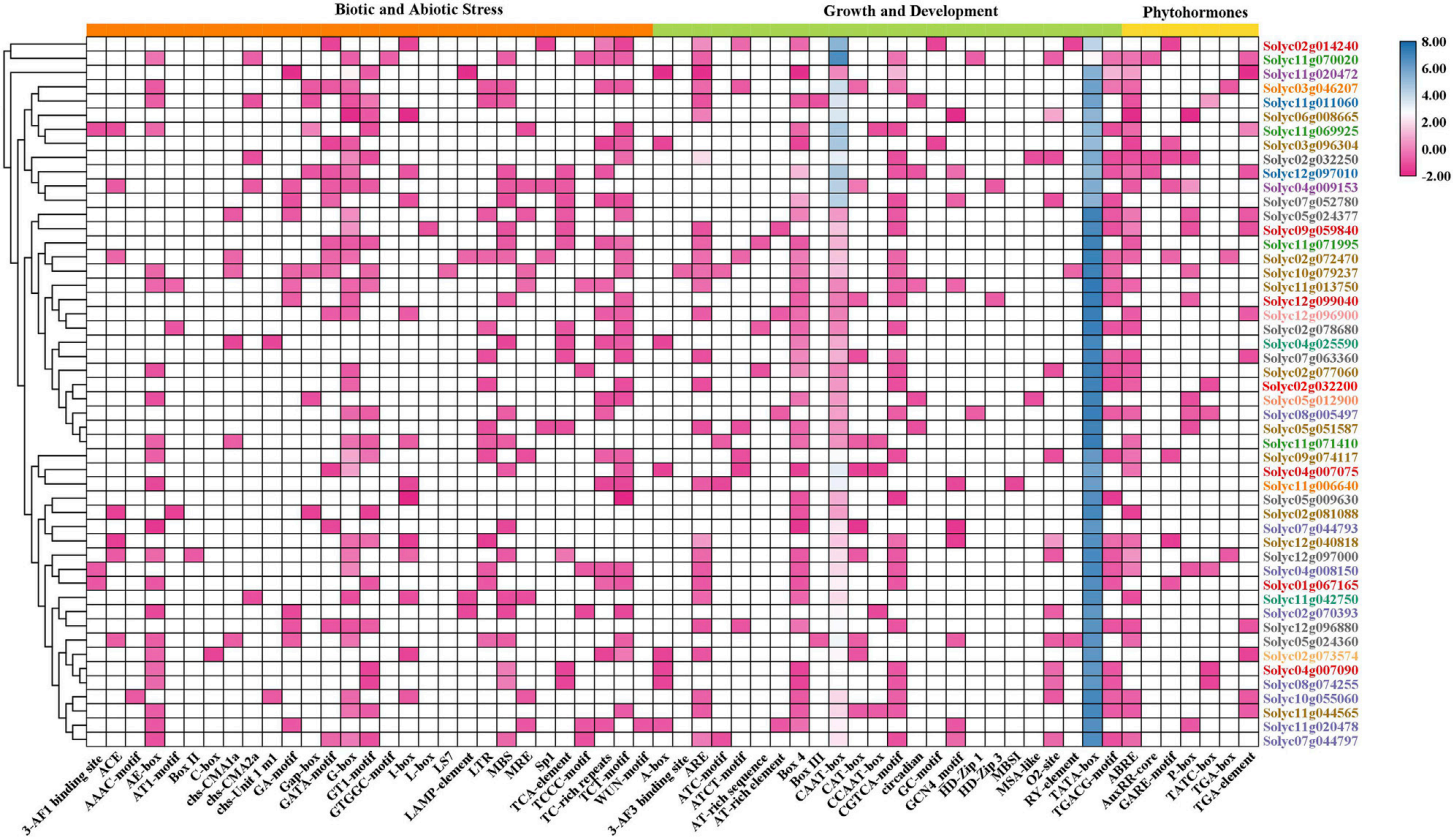
Cis-regulatory elements in the promoter regions of tomato NLRs and their distribution among biotic and abiotic stresses, growth and development, and phytohormones. The *NLR* genes are listed on the right side in different colors according to subfamily. The legend color pattern of the heatmap indicates the abundance of CREs among the *NLR* genes.

Cis-regulatory elements (CRE) present in promoter regions act as transcription start sites and function as regulatory parts of genes. To understand the regulatory role of the identified genes by promoter analysis, 1500 bp regions upstream of the promoter region were retrieved for each representative gene selected from the *NLR* subfamilies. CRE analysis revealed that 29 regulatory elements were involved in biotic and abiotic stresses, 24 in growth and development, and seven in phytohormone response (Figure 3). The heatmap demonstrated that the elements for biotic and abiotic responses, including Box-II, C-box, I-box, LS7, and 3-AF3 binding site, present in each gene’s promoter region showed medium expression levels, while the TGA box was associated with phytohormone response. The maximum abundance of TATA-box and CAAT-box was associated with *NLR* genes for growth and development.

### Phylogenetic analysis of tomato *NLR* genes with other species

**FIGURE 4:**
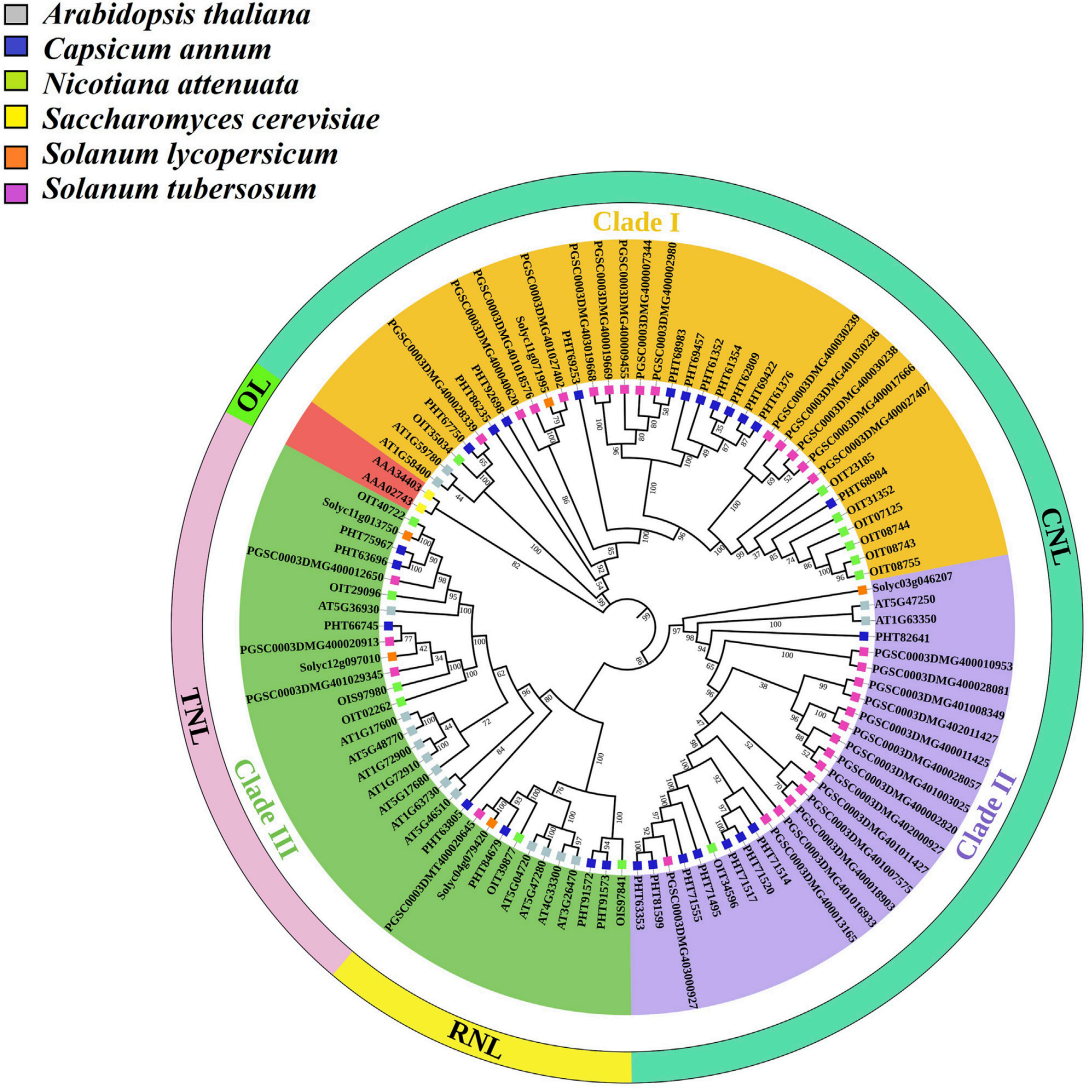
Tomato *NLR* gene orthologs in four different species (*Solanum tuberosum, Capsicum annum, Nicotiana attenuata,* and *Arabidopsis thaliana*), with yeast (*Saccharomyces cerevisiae*) as an outlier (OL) for confirmation of the bootstrap method. A phylogenetic tree was constructed with other species in MEGAX using the ML method with 1000 bootstrap replications after MSA in CLUSTALW. The bootstrap values are presented in a scale up to 100. The colored squared branch symbols show orthologs in other different species, as shown in the legend.

To understand the evolutionary relationship of tomato *NLR* genes, one representative tomato gene from each sub-family (TNL, CNL RNL, CN, N, and TN) was selected. These sequences were subjected to BlastP using threshold values of >70% similarity and E-value <1e^-5^ for the identification of orthologs in other related species, including potato, pepper, tobacco, *A. thaliana*, and yeast (selected as an outlier). According to tree topology, three major clades were further subdivided into *NLR* sub-families based on their respective domains, whereas outlier *Saccharomyces cerevisiae* sequences validated the bootstrap replication through the ML method as it appeared as an outer sub-group while the other three major clades presented according to their domain compositions (Figure 4). Clades I and II showed included CNL subfamily genes (*Solyc11g071995* and *Solyc03g046207*) and their orthologs. These clades are distributed with CNL domain-sharing genes and showed the close relationship of tomato CNL genes with other Solanaceae family members, followed by *A. thaliana*. Clade III showed two sub-clades with major subfamilies distributed with genes from TNL (*Solyc11g013750*) and TN (*Solyc12g097010*) subclasses, which were closely linked to other Solanaceae family members. This clade also contained RNL (*Solyc04g079420*) subfamily genes clustered in association with pepper and potato genes. These results suggested that the *NLR* gene subfamilies in tomato shared close ancestral relationships with other family members and was associated with the model plant (*Arabidopsis thaliana*). The orthologs in tomato might show the same pathogen interaction as in the Solanaceae family, especially in *Solanum tuberosum* (potato). Furthermore, we also assessed the evolution of the *NLR* gene family and the number of genes in other species (Supplementary Table S4).

### Synteny and Ka/Ks analysis of *NLR* genes

**FIGURE 5:**
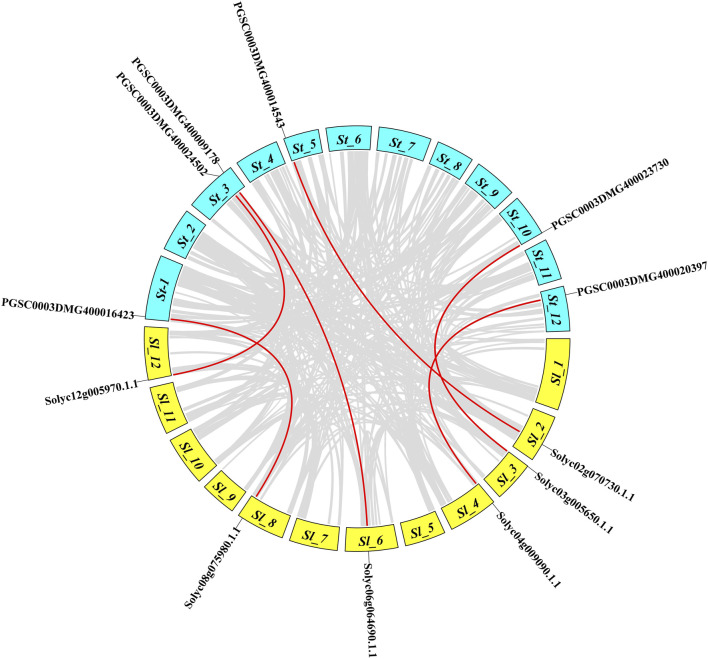
Synteny analysis of the tomato *NLR* gene family compared to potato. The genes on different circular bar blocks indicate their chromosomal positions, while the red lines represent duplicated genes that diverged between the species.

The Solanaceae family evolved 52–90 million years ago and includes potato, eggplant, tobacco, tomato, and pepper plants. To analyze the collinearity relationships among the *NLR* gene orthologs of tomatoes and potatoes, a microsynteny map was constructed. A total of six orthologs made syntenic gene pairs; for instance, tomato ortholog *Solyc02g070730* present on *Solanum lycopersicum* *Sl*-2 paired with the potato gene *PGSC0003DMG400014543* on *Solanum tuberosum* St-5 (Figure 5). *Solyc12g005970* paired with PGSC0003DMG400024502 on *St*-3, *Solyc08g075980* paired with PGSC0003DMG400016423 on St-1, *Solyc03g005650* paired with PGSC0003DMG400023730 on St-10, *Solyc04g009090* paired with PGSC0003DMG400020397 on St-12, and *Solyc06g064690* paired with PGSC0003DMG400009178 on St-3. These pairings suggested an evolutionary relationship between the tomato and potato genomes.

To understand the evolutionary origin of genes under selective pressure, synonymous and non-synonymous substitution rates (Ka/Ks) with divergence time (Mya) were calculated for the syntenic gene pairs among tomato and potato (Supplementary Table S5). Ka/Ks values <1 indicate negative/purifying selection, while values of 1 and <1 indicate neutral and positive/natural selection, respectively. All identified gene pairs showed Ka/Ks <1, suggesting that the genes were under negative or purifying selection. With the substitution rate, the divergence time of those genes ranged from 19.5 to 91.6 million years ago (Mya).

### Genotype evaluation based on the disease reactions of infected tomato leaves

The early blight disease responses of all *S. lycopersicum* genotypes (38037, 38046, 19890, and ROMA) and one wild *S. chilense* accession (19906) were analyzed by comparing the control and infected plants after treatment with fungal culture in the detached leaflet experiment (Supplementary Figure S3). Disease scoring was performed based on the affected area or disease severity percentage for genotype evaluation in response to infection (Supplementary Table S6).

### Expression profiles of tomato NLRs in transcriptomic data

**FIGURE 6:**
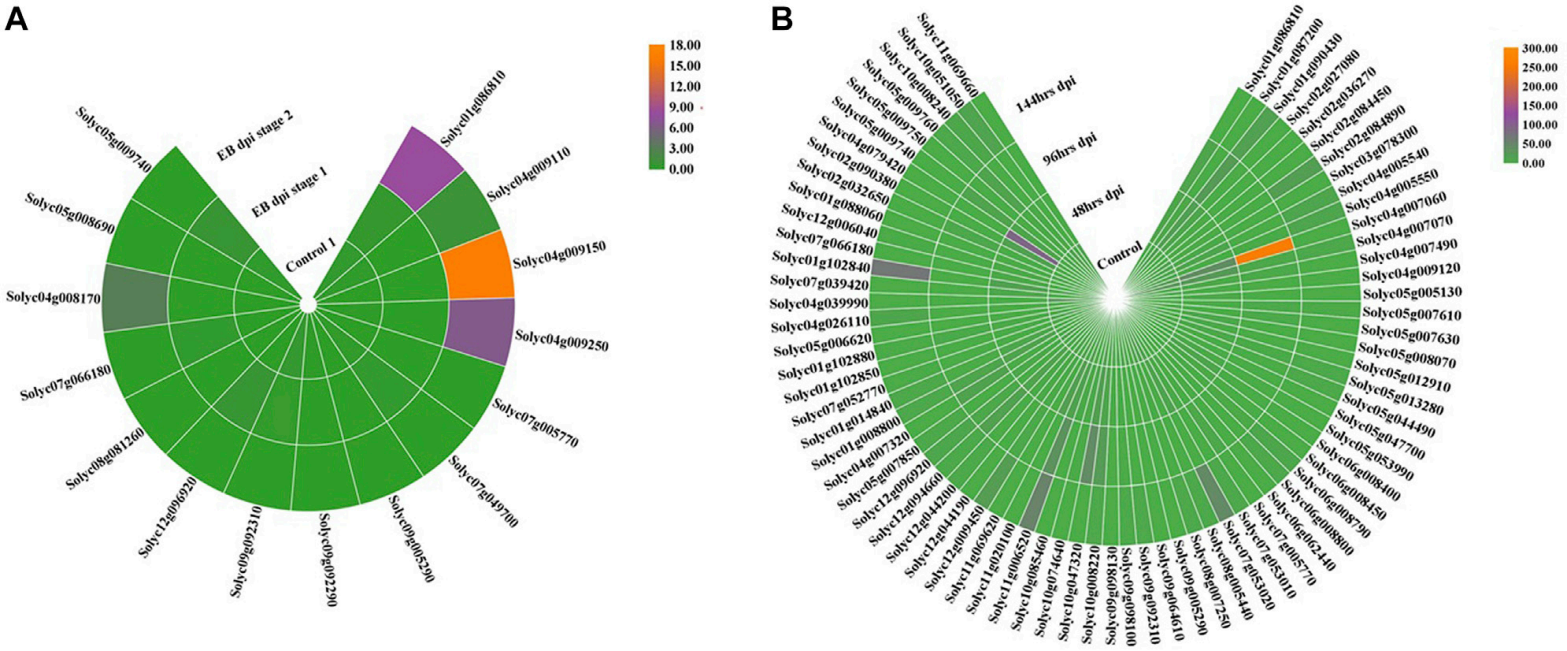
RNA-seq expression profile of DEGs from the *NLR* gene family in tomato leaf tissue under control and disease conditions in early **(A)** and late **(B)** blight shown in hours after inoculation. Orange: higher transcriptomic abundance; green: low transcription level.

To examine the expression levels of the identified *NLR* genes in tomato during fungal infection, publicly available RNA-seq data of *S. lycopersicum* were retrieved. Data on early blight (EB) and late blight (LB) were obtained from the GEO database (accession number GSE75923) and GenBank Sequence Read Archive (SRA-NCBI) (accession number SRP041501), respectively. Differentially expressed genes (DEGs) were identified against early and late blight diseases in both control and infected samples (tomato leaf tissues) (Figure 6A,B). Among the 321 NLRs, 15 for early blight and 75 for late blight were differentially expressed between control and infected leaves. These findings indicated that NLRs play significant roles in conferring tolerance to fungal blight.

*NLR* genes showed both upregulation and downregulation in leaf tissues in the control and infected leaves. In early blight disease, four genes were upregulated in EB-infected tissue compared to the control plants. Two of these genes (*Solyc01g086810* and *Solyc04g009150*) were selected for expression pattern analysis. The remaining 11 DEGs were downregulated under disease stress as compared to the controls. In late blight disease, four *NLR* genes showed differential expression (*Solyc04g009110, Solyc10g008240, Solyc04g007070, and Solyc04g007060*). These genes showed upregulation under disease (LB) inoculation in plants. The remaining genes showed lower transcriptional levels in infected leaves than in the control leaves. These findings suggested differential *NLR* behavior in late blight tolerance.

### Validation of expression levels in tomato NLRs infected by different fungal diseases by quantitative PCR

Based on the transcriptomic data, two genes (*Solyc04g009150* [*RPP13*] and *Solyc01g086810* [*RPM1*]) associated with early blight resistance and four genes (*Solyc04g007060* [*NRC4*], *Solyc10g008240* [*R1B12*], *Solyc04g007070* [*RIB16*] and *Solyc04g026110* [*Rpi*-gene]) associated with late blight resistance were selected for qPCR validation. These genes showed dynamic expression patterns in pathogen-infected plants. Between the two early blight resistance NLRs, *Solyc04g009150 (RPP13)* showed upregulation in infected tolerant wild genotype 19906 (*Solanum chilense*) and tolerant genotype 19890 (*S. lycopersicum*) plants compared to the controls.

**FIGURE 7:**
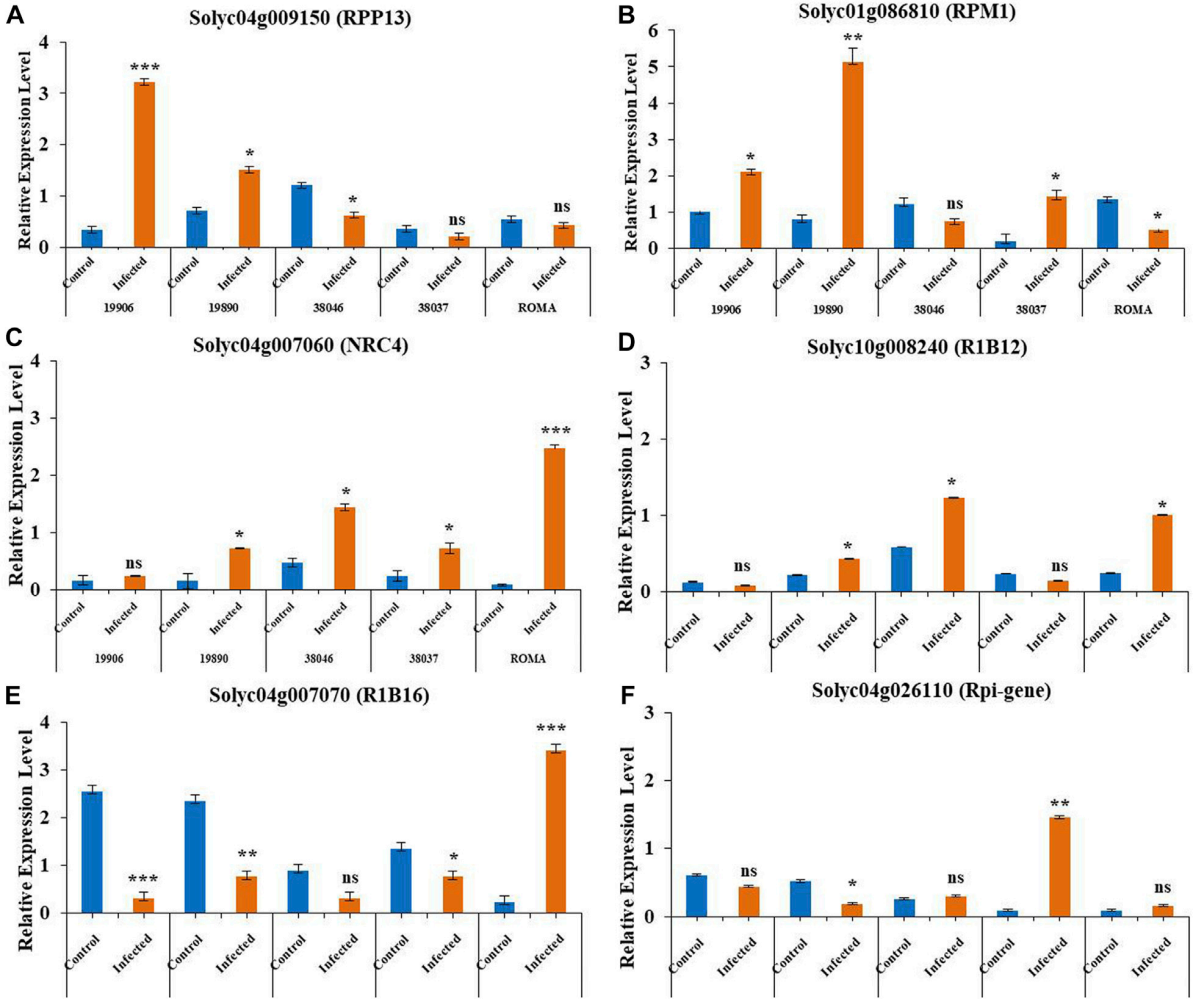
Relative expression levels of six *NLR* genes in tomato in response to fungal (early and late blight) disease condition. Three technical replicates were performed, with EF (elongation factor) as the positive control (reference gene). Light orange: infected; blue: control. *P˂ 0.05, **P ˂0.01, and ***P ˂0.001 (t-tests). Vertical lines on the bar graph: standard deviation (S.D) of three replicates.

However, among moderately susceptible genotypes, 38046 showed increased expression in the control compared to infected plants, while 38037 did not show a significant difference between control and infected plants. The susceptible genotype ROMA also showed no change in expression pattern between the control and infected samples (Figure 7A). These results suggested similar patterns of expression with the transcriptome levels of these *NLR* genes. In *Solyc01g086810* (RPM1) (Figure 7B), infected moderately tolerant genotypes 19890 19906 and moderately susceptible genotype 38037 showed similar expression patterns, in which expression levels were higher in infected plants than in the control. 38046 (moderately susceptible) and ROMA (susceptible) showed downregulation in infected plants. The expression pattern of *Solyc04g007060 (NRC4)* was also examined in susceptible, moderately susceptible, and tolerant tomato genotypes, in which 19906 (wild genotype) did not show a significant difference between control and infected plants. The remaining genotypes showed higher expression levels in plants with late blight disease than in the control plants (Figure 7C). Similarly, *Solyc10g008240* (RIB 12) expression was upregulated in response to infection compared to control plants, except for genotypes 19906 and 38037, which showed higher expression levels in control plants, although the difference was not statistically significant (Figure 7D). The inflorescence signals for *Solyc04g007070 (RIB16)* and *Solyc04g026110 (Rpi*-gene) were higher in control plants of the examined genotypes than in the infected plants, except for the ROMA and 38037 genotypes (Figures 7E,F). The expression data of these genes revealed patterns consistent with the transcriptome data. The statistical values for relative expression patterns based on ΔΔCT values in the qPCR validation of genotypes for *NLR* genes in different tomato genotypes are shown in Supplementary Table S7.

### Genotype-dependent gene expression of tomato NLRs infected by fungal diseases

The expression analysis by qPCR revealed differential expression levels. The wild genotype 19906 showed a strong increase in the expression of early blight resistance NLRs during infection. Furthermore, this genotype did not show a significant difference between control and infected plants and increased expression in the control plants for late blight resistance NLRs. A similar pattern was observed in tolerant genotype 19890. Furthermore, the moderately susceptible varieties 38046 and 38037 showed variable responses with respect to each *NLR* gene, while the susceptible variety ROMA showed significantly increased response for three genes (*Solyc04g007060, Solyc10g008240*, and *Solyc04g007070*) under disease stress compared to the control and no change in expression levels for *Solyc04g026110* and *Solyc04g009150. Solyc01g086810* showed significant responses in the control plants compared to the infected plants. These results indicated the specific regulation of tomato *NLR* genes in disease regimes and that their regulation might depend on the genetic architecture of the tomato genotypes.

### Chromosomal localization of tomato *NLR* genes and meta-QTL analysis

**FIGURE 8:**
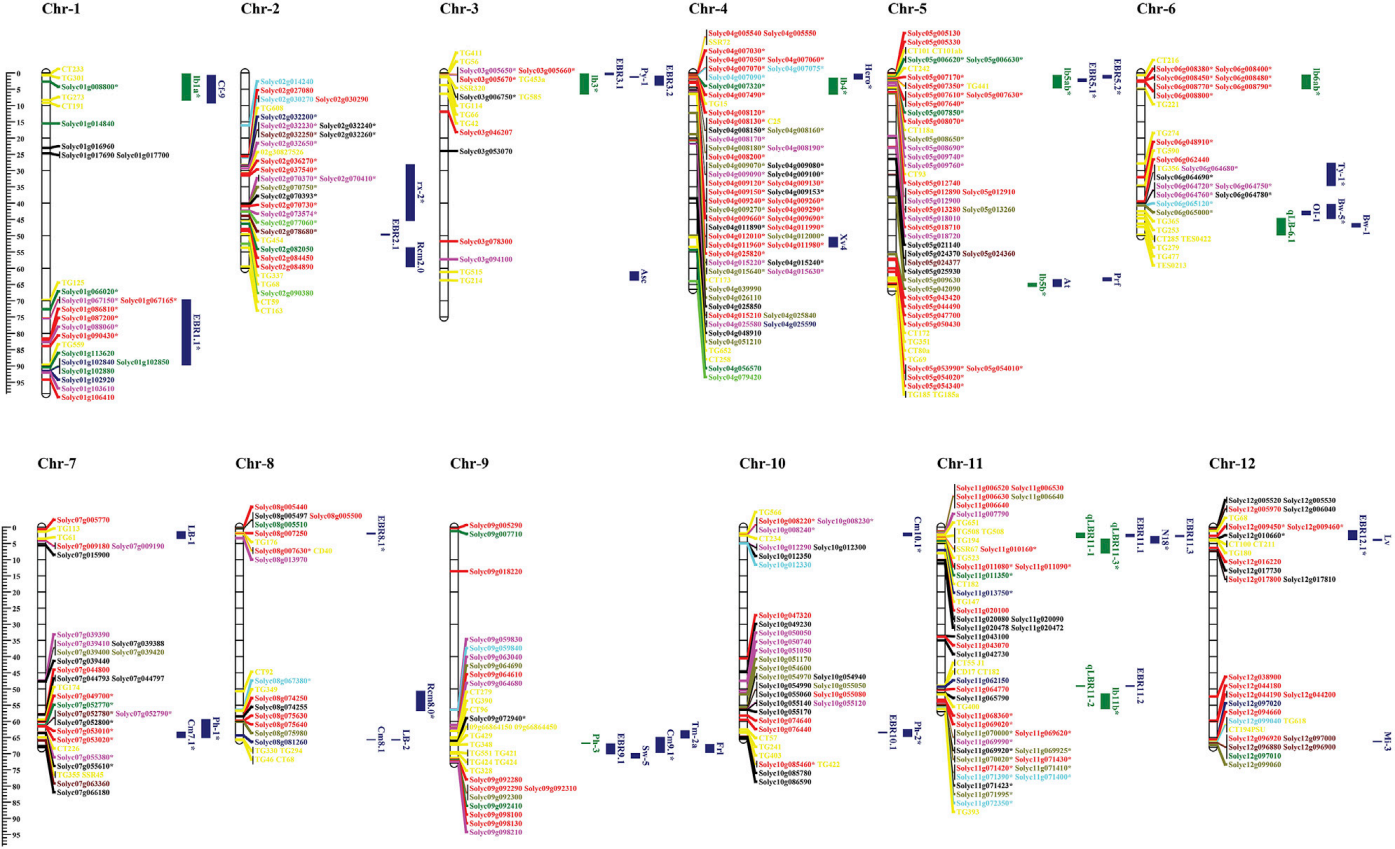
Co-localization of the number of *NLR* genes distributed on 12 chromosomes of tomato mapped with QTLs. Blue: blight resistance (EBR); green: late blight resistance (LBR). The physical positions are shown in Mbp. Gene colors indicate f *NLR* gene subfamilies in tomato. Red: CNL; dark green: TNL subfamily; dark blue: TN; bright red: NL; bright green: RNL; dark red: TIR; sky blue: LRR; mustard: CN; black: N sub-family. The markers present within QTL intervals are shown in yellow on the chromosomes. Genes within the QTLs are marked with asterisks.

The meta-QTL (MQTL) approach is used to identify the quantitative trait loci (QTLs) of complex diseases; i.e., early and late blight from previous studies. We compared these QTLs to the identified *NLR* genes to reveal the genetic regulators of these QTLs. The numbers of identified *NLR* genes were mapped against reported QTLs for fungal disease resistance and their distribution on 12 tomato chromosomes (Figure 8). The chromosomal localization of the *NLR* genes showed an uneven distribution of genes across the tomato chromosomes. For instance, genes from the CNL subfamily reside on all 12 chromosomes, while TNL genes were absent on chromosomes 3, 6, and 10. The maximum numbers of *NLR* genes were present on chromosomes 4, 5, and 11.

Meta-QTL analysis has shown that chromosome 1 harbors three QTLs, including two related to early blight (blue) and one related to late blight (green). EBR1.1 QTL contained seven genes co-localized from the CNL, TNL, and NL subfamilies. The late blight-related QTL lb1a was linked to one gene (*Solyc01g008800*) from the TNL subfamily.

Chromosome 2 included three QTLs linked to early blight resistance and one QTL linked to late blight resistance. EBR rx-2 QTL shared seventeen genes unevenly from all the categorized sub-classes.

Chromosome 3 included one late blight-related QTL and four early blight-linked QTLs. LBR QTL lb3 co-localized with four genes from the N, CN, and NL sub-families.

Chromosome 4 included two QTLs linked to early blight resistance and one QTL related to late blight resistance. The late blight-related QTL lb4 co-localized with thirty-one *NLR* genes.

Chromosome 5 included six QTLs including two QTLs related to late blight and two related to early blight resistance. QTL Lb5b co-localized with four genes belonging to the CNL subfamily, while QTL lb5ab contained 11 genes from the CNL, TNL, and NL sub-classes. Moreover, EBR QTL EBR5.1 co-localized with five genes from CNL and one TNL subfamily.

Chromosome 6 included six QTLs, two linked to the late blight resistance and four to early blight resistance. LBR QTL lb6ab co-localized with eight *NLR* genes belonging to the CNL and RNL subfamilies.

Chromosome 7 included two early blight-related QTLs and one late blight-related QTL. QTL Ph-1 was linked to late blight resistance in seven co-localized genes while. One early blight-associated QTL (cm7.1) co-localized with two genes from the NL and N sub-classes.

Chromosome 8 included four QTLs linked to early blight resistance and no QTLs associated with late blight. QTL EBR8.1 co-localized with *Solyc08g007630* belonging to the CNL sub-family, while QTL Rcm8.0 shared an interval with *Solyc08g067380* containing the LRR domain.

Chromosome 9 included one late blight-related QTL and four early blight-related QTLs. One early blight-linked QTL, Cm9.1, co-localized with *Solyc09g072940* belonging to the N subfamily.

Chromosome 10 included three MQTLs linked to early blight resistance. QTL Cm10.1 co-localized with three genes from CNL and NL subfamily, while Ph-2 co-localized with *Solyc10g085460* from the CNL subfamily.

Chromosome 11 included eight QTLs, four each linked to early blight and late blight. Among early blight-related QTLs, QTL N18 co-localized with *Solyc11g010160*, which belonged to the CNL subfamily. Among the late blight-related QTLs, qLBR11-3 co-localized with four genes; three from TNL and one from the N subfamily.

Chromosome 12 included three early blight-related QTLs. QTL EBR12.1 co-localized with three *NLR* genes: *Solyc12g009450* and *Solyc12g009460* belonging to the CNL subfamily and *Solyc12g010660* belonging to the N subfamily.

The co-localization of *NLR* genes with the early/late blight disease QTLs on different chromosomes suggests that these NLRs may be key regulators of disease resistance among the QTL regions.

## Discussion

Plants are exposed to various biotic and abiotic stresses that affect their growth and yield. Hence, plants have evolved effective immunity against different kinds of pathogens (Gull et al., 2019). Tomato is an economically important fruit crop; however, its growth is dependent on fungicide applications against fungal diseases (*Alternaria solani* and *Phytophthora infestans*), resulting in annual yield loss (Saleem et al., 2015). Resistance genes (R genes) are an important source of signal transduction pathways, which initiate plant–pathogen interactions. The R-gene family includes a large group of *NLR* (nucleotide-binding leucine-rich repeats) genes ubiquitous to plant species that are involved in disease tolerance (Ma et al., 2021). Recently, the evolution and diversification of these *NLR* gene families in numerous plant species have been elucidated based on whole-genome sequencing techniques (Zhang et al., 2016; van Wersch et al., 2020).

The current study identified 321 *NLR*-encoding genes in the tomato genome in modification to previous studies using *NLR* genes from potato and domain-based BlastP framework confirmed by SMART, Interpro, and Pfam approaches. Among these 321 genes, 211 had full-length *NLR* domains and were classified into three subfamilies (CNL, TNL, and RNL), while 110 genes were NB-coding-resistant proteins containing partial domains TN, CN, and N lacked LRR sequences with improved annotations. Our findings regarding these 321 NLRs in tomato supported the classification and distribution of *NLR* genes in the Solanaceae family (Andolfo et al., 2021).

The difference in the number of *NLR* genes between present research and earlier studies within tomato is due to improved genome annotation, selective strategies, and threshold differences for *NLR* gene identification (Seo et al., 2016; Arafa et al., 2017; Shi et al., 2021). Genome size does not correlate with the number of *NLR* genes present in various plants, as potato has a smaller genome than tomato but more NLRs due to evolution (Li et al., 2021). Tomato contains fewer NLRs than other Solanaceae family members including pepper, potato, eggplant, and tobacco, whereas potato is considered the ancient ancestor of *NLR* genes. A reduced number of *NLR* genes despite the mega genome size can occur due to the loss of a signal transduction component or ecological distribution (Liu et al., 2021).

The TNL and CNL gene ratio in tomato (*S. lycopersicum*) was approximately 1:4, consistent with the predominance of CNLs in other Solanaceae family members. The same ratio was observed in tomato’s wild species (*S. pimpenifollium*), with sequence diversity providing broad-spectrum resistance against various pathogens (Qian et al., 2017).

Diverse motif composition was observed with respect to gene subfamily, with genes in the same sub-class, i.e., the CN group, showing the same motif pattern. The identified major (P-loop, GLPL, and Kinase2) and minor (RNBS-B and RNBS-D) motifs in the conserved domain component NB-ARC were consistent with those reported by Seong et al. (2020). The *NLR* genes also showed structural diversity, with CDS, exons, and introns. No introns were present in 40% of the genes. However, alternate exons and exon shuffling may involve versatile protein formation due to the presence of non-coding regions, which also help in exon protection from changes and tend to be conserved for expression (Li et al., 2019).

Phylogenetic analysis based on sequence similarity revealed the classification of *NLR* genes into three subfamilies as reported in previous studies of NLRs in *Raphanus sativus L*. (Ma et al., 2021), *S. bicolor* (Zameer et al., 2021, and *D. rotundata* (Zhang et al., 2020) These subfamilies (CNL, TNL, and RNL) were further sub-clustered according to their functionally conserved domains. The CNL subfamily out-grouped into 12 sub-clusters, while TNL and RNL contained two sub-clusters (Andolfo et al., 2014). Two *NLR* genes, ADR1 and NGR1, with the RPW8 domain in their N-terminus region were potentially responsive against powdery mildew, a fungal disease. The RNL subfamily is considered part of the CNL and did not form a separate clade in some plants, whereas the tomato genome contained three RNL domain-encoded genes with the same motif composition (Ma et al., 2021). Tree topology contrary to this showed that the RNL clade was not embedded in CNL genes but rather presented a separate mini-cluster with a functional domain, indicating that NLRs in plants diverged into three major subclasses after originating from a common ancestor (Shao et al., 2019). With the help of ancestral developments and functional divergence, a new source of resistance can be identified, suggesting that the *NLR* gene present in the same cluster would confer resistance against a specific type of pathogen (Andolfo et al., 2013). The NLRs showed evolutionary relationships NLRs among Solanaceae family members, with a high level of conservation among Solanum species and a weak association with *A. thaliana.* These findings suggested ancestral duplication or divergence (Wu et al., 2021). An outlier, *S. cerevisiae*, was chosen to assess the reliability of the ML bootstrap replication method, as it out-grouped from the rest of the clustered genes.

Regulatory elements are involved in different responses in the promoter regions of *NLR* genes. Cis elements in *NLR* promoters showed responses to biotic and abiotic stresses compared to growth and development and phytohormones. Box-II, C-box, I-box, LS7, and 3-AF3 binding sites were observed in nearly all promoters of genes against biotic and abiotic stresses. These play key roles in plant protection against pathogens, while the TGA box is present for phytohormones. Moreover, TATA and CAAT boxes were present in maximum abundance for growth and development, consistent with results reported in previous studies (Zameer et al., 2021).

The microsynteny map constructed using *NLR* orthologs in potato and tomato occupying specific chromosomal locations comprising 6% of the tomato and potato genomes, which was consistent with the findings reported by Seong et al. (2020). Ka/Ks analysis and divergence time for syntenic gene pairs between the potato and tomato genomes suggested segmental duplicates that diverged 19–63 million years ago. The evolutionary pattern indicated that these sequence homologs underwent negative or purifying selection, as supported by previous findings (Zhang et al., 2020).

Expression validation of differentially expressed NLRs by qPCR showed strongly increased expression in tolerant and wild genotypes in early blight-resistant NLRs in a study on the induction of defense response upon disease inoculation (Jacob et al., 2013). However, the susceptible genotype ROMA showed higher expression in control than in infected plants. Genotypes with higher expression levels in the control than in infected plants support the upregulation of resistance gene pathways by indicating the downregulation of gene expression after pathogen invasion (Di Donato et al., 2017; Wang et al., 2021). In the case of late blight-resistance NLRs, *Solyc04g007060* was upregulated in infected plants compared to controls, although ROMA also showed significantly increased expression in infected plants compared to the control. In contrast, *Solyc04g007070* showed the same pattern of higher transcriptional levels in the control samples of all genotypes than in infected plants, except ROMA, which suggested the signaling pathway initiation and modulation of R-genes (Wen et al., 2020). *Solyc04g026110* expression did not differ significantly between control and infected plants except for one moderately susceptible genotype (38037) (Wu et al., 2017). *Solyc10g008240* was upregulated in the tolerant genotypes 19890 and 38046; the other genotypes did not show significant responses. The expression patterns of NLRs in the examined genotypes showed a somewhat consistent pattern with that in previous studies, except for some genotypes with higher expression in the control samples. These findings indicated that genotypes resist the effect of the pathogen by downregulating gene expression through the upregulation of resistance pathways. These include SA signaling and the PR-protein pathway, hypersensitive response, and programmed cell death, at the cost of plant growth and development (MacQueen and Bergelson, 2016). While these responses may be affected by RNA-editing sites or factors where amino acid changes affect the codon, like SNP variations are responsible for the opposite response, which may not allow gene upregulation (Wang et al., 2021). The differential expression of NLRs for disease tolerance in wild and moderately susceptible, and tolerant and susceptible cultivated genotypes, suggests the key role of the overexpression of these antifungal genes and gene pyramiding of NLRs against disease management in managing disease (Kumar et al., 2019).

The distribution of *NLR* genes on tomato chromosomes observed in the present study was consistent with that reported by Shi et al. (2021). The maximum number of genes was distributed on chromosomes 4, 5, and 11. A meta-QTL analysis to obtain insight into the genomic organization of late blight resistance loci based on the closest flanking markers revealed that major QTLs co-localize with mapped *NLR* genes on tomato chromosomes (Khahani et al., 2021). These findings suggested that these loci might be involved in resistance to late blight diseases. The late blight-related resistance QTL lb4 co-localized with the *Solyc04g007060* gene, an expression pattern that was validated by qPCR. This gene determines the induction of defense response upon pathogen invasion, suggesting the role of *NLR* genes in disease tolerance and function as positive regulators of resistance (Danan et al., 2011). The identified QTLs can be further fine-mapped to identify genes within promising regions resistant to diseases (Kumar and Nadarajah, 2020).

NLRs are believed to co-evolve rapidly with pathogens, requiring improved understanding of the molecular mechanisms of disease resistance and the development of resistant cultivars with improved breeding techniques.

## Conclusion

The genome-wide identification of NLRs is a potential approach for resistance gene cloning and functional characterization. Our results extend the understanding of the abundance and diversity of *NLR* genes in tomato, which may inform gene pyramiding after functional validation. Furthermore, the molecular mechanisms of disease resistance, the development of resistant cultivars, and the over-expression of these NLRs through CRISPR technology may be essential approaches to disease tolerance.

## Data Availability

Publicly available datasets were analyzed in this study. The names of the repositories and accession number(s) can be found in the article/Supplementary Material. All other data in the study are available on request from the corresponding authors.
