# Supplementary material for: Genome-wide characterization of the NLR gene family in tomato (Solanum lycopersicum) and their relatedness to disease resistance
Source: Front Genet. 2022 Dec 5;13:931580. doi: 10.3389/fgene.2022.931580 (PMC9760929; doi:10.3389/fgene.2022.931580)
Supplement: Supplementary file 3 [file Table1.DOCX]

**Supplementary Table S1:** List of primers used during qRT-PCR

| **No.** | **Expression Primers** | **Primer’s (F/R) sequences** |
| --- | --- | --- |
| 1. | *Rpi*-gene (*Solyc04g026110*)-F | 5’-CATCGACATGGAGAAGACAT-3’ |
|  | *Rpi*-gene (*Solyc04g026110*)-R | 3’-CACGACTTGGTTCAGCATAG-5’ |
| 2. | *RPM1* (*Solyc01g086810*)-F | 5’-CGATGCTGCAAATGATCGCA-3’ |
|  | *RPM1* (*Solyc01g086810*)-R | 3’-CTCCACGAGCCAACCGATTA-5’ |
| 3. | *RPP13* (*Solyc04g009150*)-F | 5’-TCGTCTCAAGGCTTGTGCTT-3’ |
|  | *RPP13* (*Solyc04g009150*)-R | 3’- GCGAGCGATGTCCATGATTT-5’ |
| 4. | *NRC4* (*Solyc04g007060*)-F | 5’- CTTCAGGCAACCCCTGTTCT-3’ |
|  | *NRC4* (*Solyc04g007060*)-R | 3’-AACCCGACCACAGCATCATT-5’ |
| 5. | *R1B16* (*Solyc04g007070*)-F | 5’-TGGAGTACTGAGGCATGGGA-3’ |
|  | *R1B16* (*Solyc04g007070*)-R | 3’- GGGCAGTTGACATAATCCGC-5’ |
| 6. | *R1B12* (*Solyc10g008240*)-F | 5’- TGATGCTCTACCAACACGGG-3’ |
|  | *R1B12* (*Solyc10g008240*)-R | 3’- GAAGGAGGCCCTATAAGCCG-5’ |
| 7. | *EF1* (control)-F | 5’-GGTATTGACAAGCGTGTTAT-3’ |
|  | *EF1* (control)-R | 3’-TCAATGGTGATACCACGCTCA-5’ |

**Supplementary Table S2:** Distribution of 321 *NLR* genes into nine sub-classes according to domain

| **NLRs Sub-families** | **No. of Genes** |
| --- | --- |
| CC-NB-LRR | 123 |
| TIR-NB-LRR | 21 |
| RPW8-NB-LRR | 3 |
| NB-LRR | 48 |
| CC-NB-ARC | 35 |
| NB-ARC | 57 |
| TIR-NB-ARC | 9 |
| LRR | 16 |
| TIR | 9 |
| **Total** | **321** |

**Supplementary Table S3: Tomato *NLR* gene features and Ids with domain Sub-classes (Excel sheet provided).**

**Supplementary Table S4: NLRs in other species**

| **NLRs** | **specie** | **References** |  |
| --- | --- | --- | --- |
| 225 | *Raphanus sativus* | Ma et al., 2021 | dicot |
| 164 | *A.thaliana* |  | dicot |
| 212 | *B.rapa* |  | dicot |
| 244 | *B.olerecea* |  | dicot |
| 468 | barley | Li et al., 2021 | monocot |
| 307 | *N.benthimiana* | Seong et al., 2021 | dicot |
| 1331 | *S.tuberosum* | Andolfo et al., 2013 | dicot |
| 458 | *Oryzae sativa* | Seo et al., 2016 | monocot |
| 95 | *Sorghum bicolor* |  |  |
| 991 | *Capsicum annum* | Seong et al., 2020 | dicot |
| 330 | *S. pimpinellifolium* | Seong et al., 2021 | dicot |
| 303 | *S. cheesmaniae* |  | dicot |
| 220 | *S. pennelli* | Araujo et al, 2020 | dicot |
| 57 | *Cucumis sativus* | Wei et al., 2016 | dicot |
| 198 | *Arabidopsis lyrata* | [Zhang et al., 2016](https://onlinelibrary.wiley.com/action/doSearch?ContribAuthorRaw=Zhang%2C+Yan-Mei) | dicot |
| 127 | *Capsella rubella* |  | dicot |
| 88 | *Thellungiella salsuginea* |  | dicot |
| 51 | *C. papaya* |  | dicot |

**Supplementary Table S5:** Ka/Ks ratio and divergence time calculation

| **Seq_1** | **Seq_2** | **Ka** | **Ks** | **Ka/Ks** | **Divergence Time (Mya)** |
| --- | --- | --- | --- | --- | --- |
| Solyc12g005970 | PGSC0003DMG400024502 | 1.058073 | 1.886553 | 0.5609 | 62.8 |
| Solyc08g075980 | PGSC0003DMG400016423 | 0.276883 | 0.585382 | 0.4729 | 19.5 |
| Solyc02g070730 | PGSC0003DMG400014543 | 1.146335 | 1.600283 | 0.7163 | 53.3 |
| Solyc04g009090 | PGSC0003DMG400020397 | 1.264005 | 2.749188 | 0.4598 | 91.6 |

**Supplementary Table S6:** Genotype evaluation upon disease inoculation and their reaction

| **Tomato genotypes** | **Lesion size ± S.E(cm)** | **Affected area (%)** | **Disease score** | **Disease reaction** |
| --- | --- | --- | --- | --- |
| 19890 | 22±0.005 | 22 | 2 | Moderately resistant |
| 19906 | 25±0.004 | 25 | 2 | Moderately resistant |
| 38046 | 50±0.04 | 50 | 3 | Moderately Succeptible |
| 38037 | 50±0.06 | 50 | 3 | Moderately Succeptible |
| ROMA | 83±0.13 | 83 | 5 | Highly Succeptible |

Disease scale

0-10% affected area HR

11-25% affected area MR

26-50% affected area MS

51-75% affected area S

76-100% affected area HS

**Suplplemtary Table S7:** Relative expression pattern demonstrated based on ΔΔCT values in qPCR validation of genotypes for *NLR* genes (Excel sheet is provided).

**
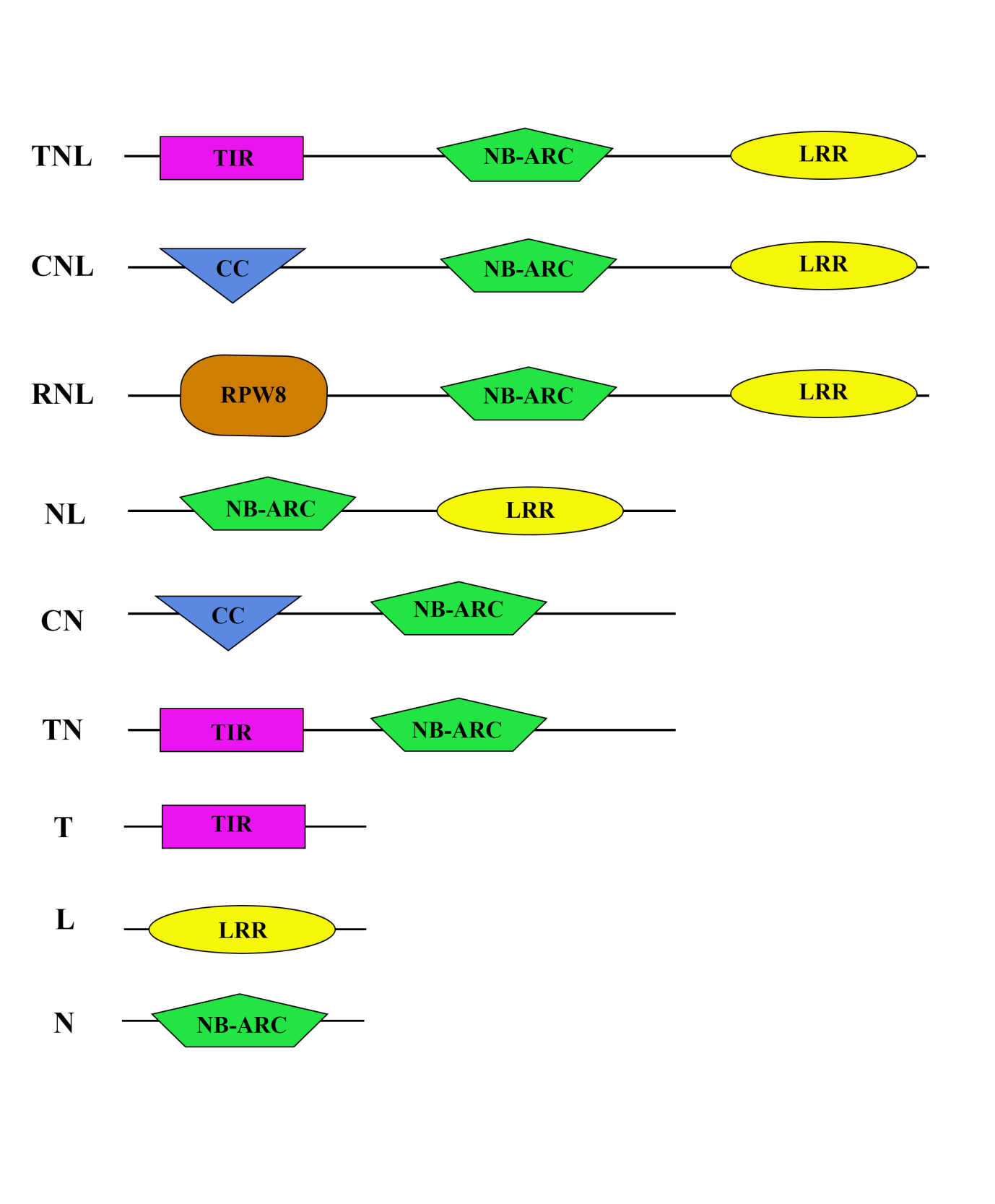
**

**Supplementary Figure S1:** Symbolic structures of *NLR* genes domain classified in nine different sub-classes represented with different shapes and colors.


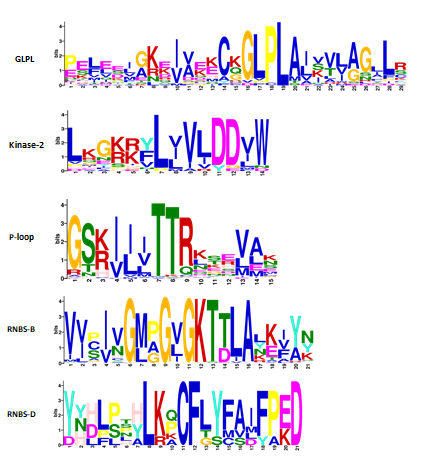


**Supplementary Figure S2**: Conserved domain NB-ARC major and minor motif logos.

**
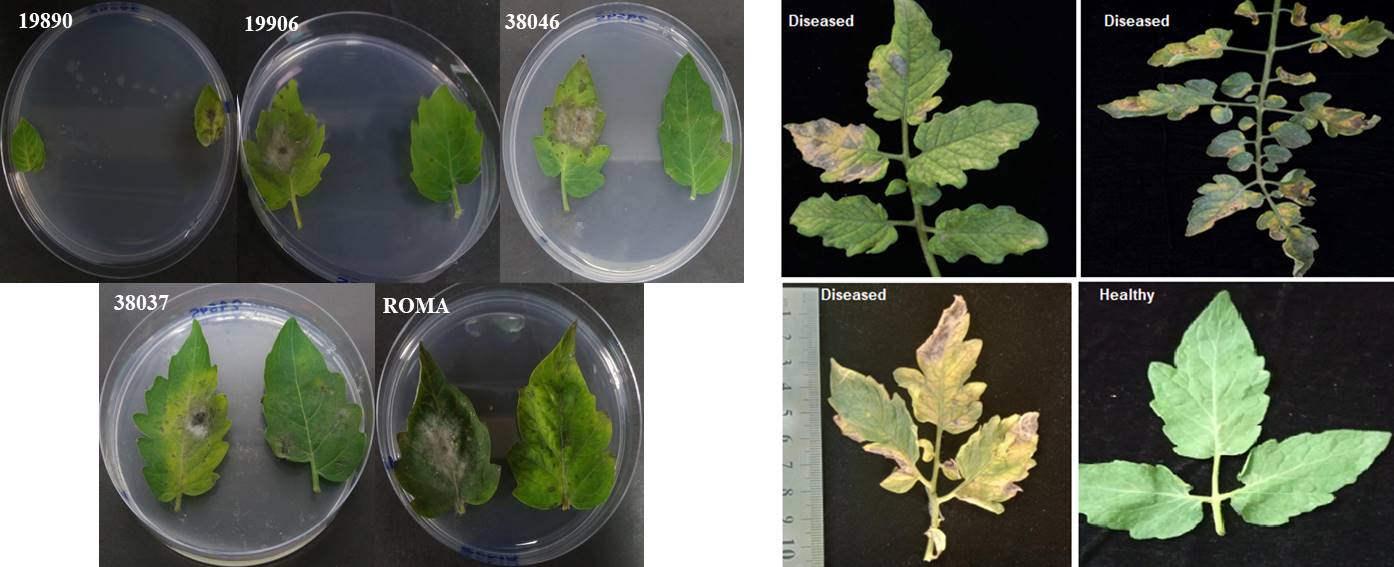
**

**Supplementary Figure S3:** Genotype evaluation after fungal disease inoculation through comparison of control and infected leaves from detachted leaflet assay.
